# In Situ Synthesis of Organic Polymer–Inorganic Nano ZnO Core–Shell Structured Sizing Agents and Their Effect on Carbon Fiber Interfaces and Composite Properties

**DOI:** 10.3390/polym17060773

**Published:** 2025-03-14

**Authors:** Wen Liu, Mudasir Ahmad, Pengfei Song, Qianli Fang, Qingchao Li, Guoqing Huang, Chuncai Yang

**Affiliations:** 1 Institute of Catalysis for Energy and Environment, College of Chemistry and Chemical Engineering, Shenyang Normal University, Shenyang 110136, China; 18241394119@163.com (W.L.); 18698785870@163.com (P.S.); 17863521569@163.com (Q.F.); 15945315056@163.com (Q.L.); 2 School of Chemistry and Chemical Engineering, Northwestern Polytechnical University, Xi’an 710072, China; mirmudasirv@nwpu.edu.cn; 3 Jilin Qianren Innovative Materials Co., Ltd., Jilin 132101, China

**Keywords:** in situ synthesis, organic polymer–inorganic nano ZnO core–shell structure composite, sizing agent, mechanical interlocking, anti-pressure resistance

## Abstract

Sizing agents are essential to address the increasing demands of enhanced carbon fibers (CFs), where increasing interfacial adhesion and the analysis of mechanical properties are achieved for critical engineering applications. In this work, five types of self-emulsifying sizing agents, featuring organic polymer–inorganic nano zinc oxide (ZnO) core-shell structures with varying crosslinked polymer densities in the core, were synthesized using self-emulsifying technology through a one-pot, in situ synthetic process. This study revealed that these sizing agents exhibited a uniform particle size distribution within the range of 100–200 nm, along with excellent storage stability and thermal stability up to 300 °C. The optimized sizing agent significantly enhanced the surface properties of CFs, achieving a surface roughness of 6.04 nm and a surface energy of 74.81 mJ/m^2^. Moreover, the interlaminar shear strength (ILSS) and flexural strength of CF/epoxy resin (EP) composites modified with the synthesized sizing agent increased by 86% and 86.43%, respectively, compared to unoptimized composites. These improvements in mechanical properties are attributed to enhanced stress transfer at the CF/EP interface, facilitated by the interlocking mechanism of the nano ZnO particle shell and the superior anti-pressure resistance provided by the crosslinked organic polymer core.

## 1. Introduction

With the rapid development of technology and a growing industrial demand, high-performance fibers have become a significant research subject in the global materials science community. These materials have been widely used in automotive lightweight structures, aerospace, and other fields due to their high specific strength and specific modulus [1,2,3]. However, due to the smooth surface of the carbon fiber (CF) and poor wettability, the bonding strength between the CF and the resin matrix is low, ultimately affecting the comprehensive mechanical properties of CF-reinforced resin composites [4,5]. The adhesion between the CF and the resin matrix can be improved by modifying the CF surface through chemical grafting [6], nanoparticle filling [7], electrochemical deposition [8,9], and sizing methods [10]. The sizing method has become the primary choice in CF production due to its easy operation and energy-saving advantages [11]. Among numerous sizing agents, self-emulsifying sizing agents have received widespread attention [12,13]. Self-emulsifying sizing agents can be realized using chemical modification to introduce hydrophilic groups into the resin molecular segments, making them amphiphilic macromolecules that can be emulsified without an extra emulsion agent [14,15,16]. Yao et al. [17] used octadecylamine and polyetheramine to modify epoxy resin to prepare self-emulsifying sizing agents and investigated the effect of the hydrophilic–oleophilic ratio on the interfacial properties of the sized CF; when the hydrophilic–lipophilic ratio of octadecylamine and polyetheramine was 19:1, the interfacial shear strength (ILSS) was maximally improved. In order to further enhance the ILSS, researchers have found that sizing agents modified with nanoparticles can achieve high surface roughness and mechanical interlocking in the interface phase, making them a hot research topic in interface enhancement [18,19]. Geng et al. [20] reported an in situ synthetic nano SiO_2_ modified and self-emulsifying sizing agent with a core–shell structure which enhanced interfacial adhesion between the sized carbon fiber and the EP through the interlocking mechanism between nanoparticles, thereby significantly improving the ILSS. However, in previous studies on nano particle-modified sizing agents with core–shell structures, the nano inorganic particles were distributed on the core and the organic components on the shell [21,22]. There were no reports on reverse core–shell structure-modified sizing agents with inorganic particles on the shell and organic components on the core, and there were even fewer studies on the influence of the elastic modulus of the core–shell nano particle on the interfacial adhesion between the sized carbon fiber and the resin matrix through the interlocking mechanism.

In this study, in situ synthetic organic polymer–inorganic nano ZnO core–shell sizing agents with different organic crosslinked polymer contents inside the core were reported. The elastic modulus of the core–shell nano particles will be tested by atomic force microscopy (AFM) and optimized through the crosslinked polymerization of MMA inside the core. The strong compressive resistance imparted by the crosslinked organic polymer core granted the ILSS and flexural strength of the CF/EP composites to be increased by 86% and 86.43%, respectively, compared to those of the unoptimized composites in which its CF was sized by a commercial sizing agent.

## 2. Experiment

### 2.1. Material

Commercial carbon fibers (CF T400) were supplied by Jilin Chemical Fiber Group Co., Ltd., Jilin, China. Epoxy resin (E-1NT) and polyetheramine (PEA, molecular weight 1000) were provided by Jilin Qianren Innovative Materials Co., Jilin, Ltd., China. Epoxy resin (AG-80) and 4,4′-diaminodiphenyl sulfone (DDS, ≥99%) were obtained from Guangzhou Taixin Science & Technology Co., Ltd., Guangzhou, China. Octadecylamine (ODA, 99%), Ethylene glycol monoallylether (2-Allyloxyethanol, EGMAE, 98.0%), methyl methacrylate (MMA, AR 99%), and Azo-bis-isobutryonitrile (AIBN, AR, 99%) were purchased from Aladdin Co., Ltd., Shanghai, China. Acetic acid (AR, 99.5%) was acquired from Tianjin Damao Chemical Reagent Factory, Tianjin, China. Zinc Acetylacetonate Hydrate (ZAA) was sourced from Shanghai Liming Chemical Co., Ltd., Shanghai, China and Acetylacetone was obtained from Tianjin Ruiting Chemicals Co., Ltd., Tianjin, China.

### 2.2. Synthesis of Amine-Modified Epoxy Resin

**Figure 1:**
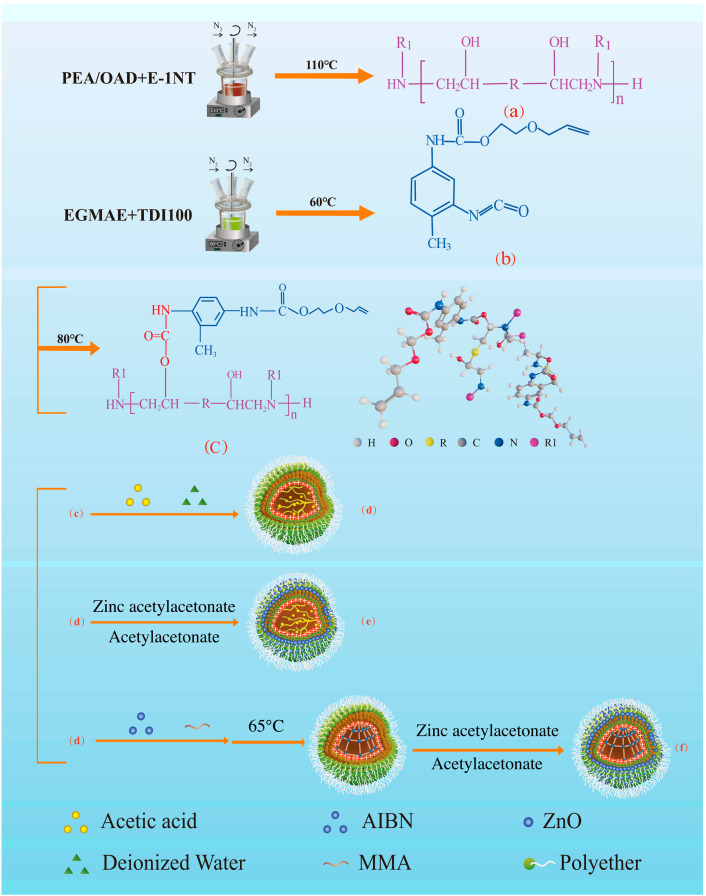
Schematic and preparation route of the five self-emulsifying organic polymer–inorganic nano ZnO core–shell sizing agents with different cross-linked polymer extents inside the core (S1 to S5). (**a**) Amine-Modified Epoxy Resin, (**b**) the Intermediate of Isocyanate Containing Allyl Groups, (**c**) Amine-Modified Epoxy Resin Containing Allyl Groups on Its Main Chain, (**d**) the Self-Emulsifying Cationic Polymer Emulsion of AEPA, (**e**) the Self-Emulsifying Nano ZnO Particle-Modified Sizing Agent and (**f**) Organic Polymer–Inorganic ZnO Core–Shell Sizing Agents.

As illustrated in Figure 1a, ODA and PEA (9:1 molar ratio) were reacted with an appropriate amount of epoxy resin E-1NT under a nitrogen atmosphere at 110 °C for 3 h to prepare an amine-modified epoxy resin with a molecular weight of 5000 via the epoxy ring-opening reaction. The chemical structural formula is shown in Figure 1a [17].

### 2.3. Synthesis of the Intermediate of Isocyanate Containing Allyl Groups

Toluene-2,4-diisocyanate (TDI100), containing two NCO groups with different reactivity, was placed in a four-necked flask that had been dried and purged with dry nitrogen. An equal molar amount of EGMAE was added and reacted at 60 °C to graft allyl groups onto the structure of TDI100 to prepare the intermediate of isocyanate containing allyl groups, as shown in Figure 1b [23].

### 2.4. Synthesis of Amine-Modified Epoxy Resin Containing Allyl Groups on Its Main Chain

The intermediate and the amine-modified epoxy resin were then placed in a four-necked flask equipped with nitrogen inlet and outlet ports and a mechanical stirrer. The mixture was gradually heated to 80 °C and held at this temperature for 2 h to produce an amine-modified epoxy resin containing allyl groups on its main chain (AEPA), as shown in Figure 1c [24].

### 2.5. Preparation of the Self-Emulsifying Cationic Polymer Emulsion of AEPA

AEPA was diluted with an appropriate volume of acetone, followed by the neutralization of the added acetic acid to form a tertiary amine salt (cationic polymer). The resulting salt was then dispersed by adding water to form a cationic polymer emulsion, as shown in Figure 1d. Finally, distilled water was added to dilute the cationic polymer emulsion to a 20% solids content.

### 2.6. In Situ Synthesis of the Self-Emulsifying Nano ZnO Particle-Modified Sizing Agent

ZAA dissolved in acetylacetone was added into the cationic polymer emulsion to prepare the nano ZnO particles-modified sizing agent containing 5% ZnO (based on the total weight of the sizing agent emulsion), resulting in the particle structure depicted in Figure 1e: the nano ZnO particles are on the shell and the organic polymer containing the allyl group are inside the core (the nano ZnO particles-modified sizing agent containing 5% ZnO is denoted by S0).

### 2.7. In Situ Synthesis of Organic Polymer–Inorganic ZnO Core–Shell Sizing Agents

As shown in Figure 1f, five ratios of MMA (3:1, 2:1, 1.5:1, 1:1, 1:1.5) and AIBN were added to the cationic polymer emulsion (Figure 1f) under sealed conditions and stirred for 12 h to stabilize the emulsion. Then, the emulsion mixture was degassed under nitrogen for 2 h, followed by polymerization at 65 °C under a nitrogen atmosphere for 3 h. Finally, a solid organic crosslinked polymer was formed as a core due to the crosslinking polymerization of MMA with the amine-modified epoxy resin containing allyl groups. After cooling to room temperature, ZAA dissolved in acetylacetone was added and stirred for 12 h to prepare a ZnO nanoparticle shell covered on the surface with the organic crosslinked polymer core which contained 5% ZnO. Finally, the emulsion was then raised to 75 °C and held for 2 h to remove the solvent, yielding the five types of organic polymer–inorganic ZnO core–shell sizing agent (S1, S2, S3, S4, S5) shown in Table 1. 

### 2.8. Preparation of Sized CF

The Soxhlet apparatus was used to desize commercial CF with refluxing in acetone for 24 h. The desized CF was then washed with distilled water and dried at 110 °C for 2 h before being cooled and stored for subsequent use. The six types of organic polymer–inorganic nano ZnO core–shell sizing agents (S0, S1, S2, S3, S4, S5) were diluted with deionized water to achieve a 1% solids content. Under the traction of the motor, the desized CF passed through the impregnating tank at a speed of 1 cm/s. Finally, five types of sized CFs (CFs0 to CFs5) impregnated with the sizing agents S0 to S5 were then dried at 110 °C for 1.5 h.

### 2.9. Preparation of CF/EP Composites

For the epoxy curing system, the mass ratio of AG-80 to DDS was 10:4. The resin matrix was melted at 135 °C until a transparent solution was formed, and then impregnated into unidirectional CFs, which were secured on a metal frame to maintain the orientation of the unidirectional CFs. The resin-impregnated unidirectional carbon fibers were placed into a mold that was preheated and treated with a release agent at 135 °C. After closing the mold with bolts, the CF/EP composite was prepared by placing the closed mold in an oven at 135 °C for 90 min and 180 °C for 120 min, followed by natural cooling to room temperature. Finally, CF/EP composite material samples (CF0/EP-CF5/EP) were prepared for ILSS testing based on the mold dimensions of (200 ± 1 mm) × (6 ± 0.2 mm) × (2 ± 0.2 mm) and named separately. The dimensions of the samples and the methods are shown in Figure S1.

### 2.10. Characterizations

The Fourier Transform Infrared (FT-IR) spectra of amino-modified epoxy, AEPA, intermediate, and organic polymer–inorganic nano ZnO core–shell sizing agents were recorded using a TENSOR II spectrometer (Bruker, Germany) to identify the chemical functionalities present in these materials. The particle size and the distribution of the sizing agents were determined using dynamic light scattering (DLS) (Malvern, Gemany) to ensure the homogeneity and stability of the dispersions. The microstructure of the sized carbon fiber surfaces was observed and analyzed using a HITACHI S-480 scanning electron microscope (Japan) (SEM). The core–shell structure of the sizing agents was examined using a Talos F200 transmission electron microscope (TEM) (Thermo Fisher, USA) to visualize the internal architecture of the particles. The surface roughness of both unsized and sized carbon fibers with different organic core contents was observed and analyzed using atomic force microscopy (AFM) over a 1 µm × 1 µm area (Dimension Icon, Bruker, Germany). The thermal stability and sizing percentages of carbon fibers with different organic core contents were tested and analyzed using a Netzsch TG209F3 thermogravimetric analyzer (Germany). The sizing agent samples were vacuum-dried at 110 °C for 12 h prior to analysis. The sized carbon fiber samples were analyzed in alumina crucibles from 20 °C to 500 °C under a nitrogen atmosphere to evaluate their thermal behavior and determine the extent of the sizing agents. The hydrophobicity of the carbon fibers was characterized using a DataPhysics contact angle analyzer. The changes in the contact angles of deionized water and diiodomethane on the carbon fiber surfaces with different degrees of crosslinking were analyzed. Each sample was tested three times, and the average values were reported. The surface energy of the carbon fibers could be calculated according to the Owens–Wend–Rabel–Kaelble (OWRK) method and Young’s equation [25] which are attached in S1.4. The interlaminar shear strength (ILSS) and flexural strength of the CF/EP composites were tested using an Instron 34TM-30 universal tensile tester. In accordance with the international standard “Test method for shear strength of polymer based composite short beams” (ISO 14125:1998 (E)), the three-point short beam method was used for testing. The average values of five tests were measured. The detailed steps are reported in Supplementary Materials S1.1–S1.3.

## 3. Results and Discussion

### 3.1. Chemical Structure

**Figure 2:**
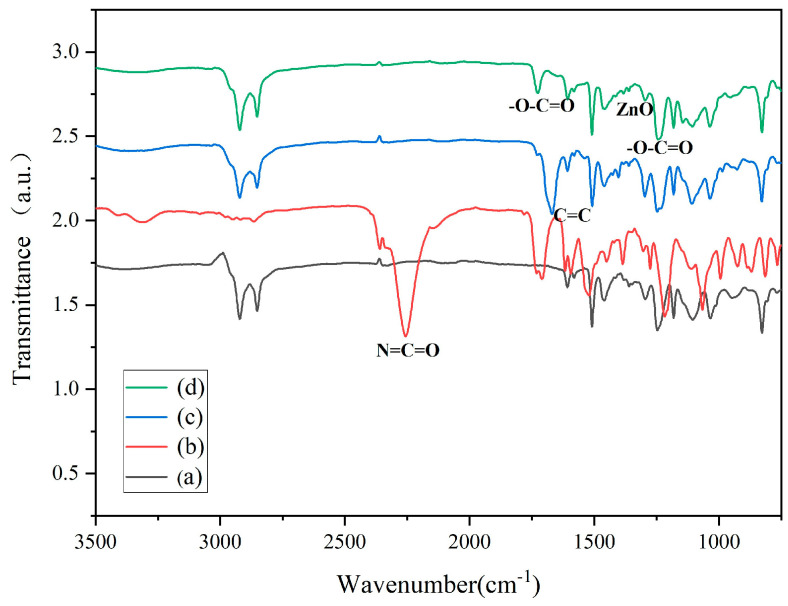
The FTIR spectrum of (a) amino-modified epoxy; (b) intermediate; (c) amino-modified resin containing allyl groups; (d) organic polymer–inorganic nano ZnO core–shell sizing agent.

Figure 2 shows the FTIR of amino-modified epoxy (a), intermediate (b), amino-modified resin containing allyl groups (c), and organic polymer–inorganic nano ZnO core–shell sizing agent (d). The FTIR spectra of amino-modified epoxy (a) was consistent with a previous report [26]. In curve (b), the presence of the characteristic peak for N=C=O at 2255 cm^−1^ and the C=C stretching vibration at 1617 cm^−1^ suggest that one of the N=C=O groups of TDI100 reacted with the -OH groups of ethylene glycol monoallyl ether, and the other N=C=O group of TDI100 remained un-reacted to form an intermediate (b) [24]. In curve (c), the absence of the characteristic peak for N=C=O at 2255 cm^−1^ indicates that the N=C=O group of intermediate (b) reacted with the -OH group of amino-modified epoxy (a) to generate an amino-modified resin with a double bond (c) [27]. In the curve (d), the disappearance of the C=C stretching vibration peak, the presence of peaks at 1240 cm^−1^ and 1727 cm^−1^ representing the -O-C=O group [20], and the Zn-O stretching vibration peak at 1380 cm^−1^ indicate that the amino-modified resin with the double bond (c) established a co-polymerization with MMA, the ZnO formed, and the organic polymer–inorganic nano ZnO core–shell sizing agent had been synthesized successfully.

### 3.2. Core–Shell Structure, Particle Size, and Storage Stability

**Figure 3:**
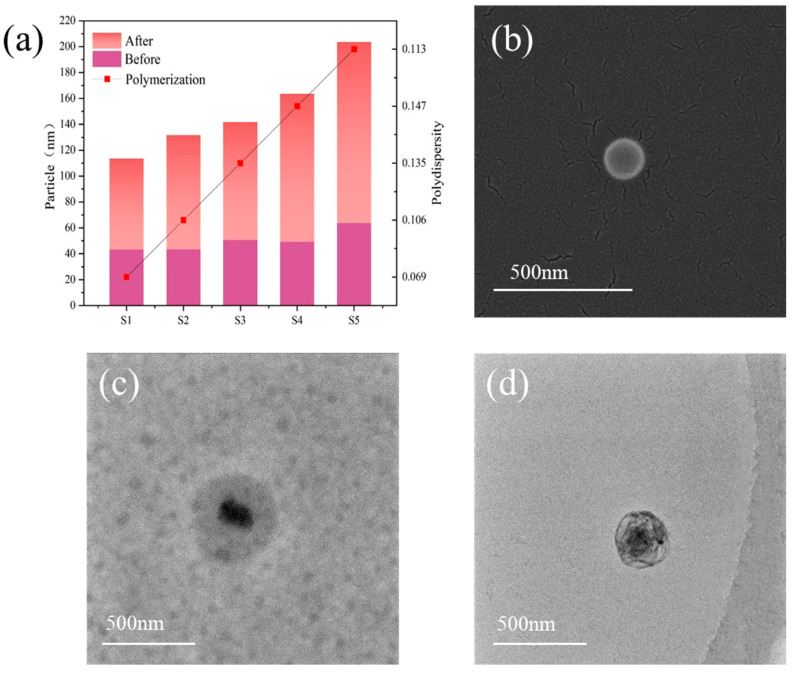
The particle size and its PDI in S1 to S5 (**a**); SEM images of S4 (**b**); TEM images of S4 (**c**); TEM images of S4 before zinc acetylacetonate was added and inorganic ZnO formed on its shell (**d**).

Figure 3 displays the particle size and particle size distribution (PSD) in the sizing agent emulsions from S1 to S5, tested by DLS. After zinc acetylacetonate was added and inorganic nano ZnO formed on the shell, all of the particle sizes increased; the average particle sizes in S1 to S5 increased linearly with the content of MMA. They are 70.35 nm, 88.06 nm, 91.04 nm, 114.3 nm, and 139.8 nm, respectively, indicating that the organic polymer cores increase their particle sizes with MMA. The polydispersity index (PDI) values of S1 to S5 were all smaller than 0.25, indicating good monodispersity [28]. The above results demonstrate that the method proposed in this study was successfully deployed to synthesize organic polymer–inorganic nano ZnO core–shell sizing agents with varying amounts of organic crosslinked polymer inside the cores.

S4 was the representative organic polymer–inorganic nano ZnO core–shell sizing agent chosen to be studied by scanning electron microscopy (SEM) and transmission electron microscopy (TEM). Figure 3b shows the SEM image; its size was 125 nm, close to the test results from DLS. The TEM images in Figure 3c clearly show the core–shell structure, Figure 3d is a TEM image of S4 before the zinc acetylacetonate was added and inorganic nano ZnO formed on its shell. Obviously, the diameter of the core–shell sizing agent becomes larger after the inorganic ZnO forms on the shell. The SEM and TEM results are consistent with the previous particle size measurements from DLS, confirming the successful synthesis of the target product.

**Figure 4:**
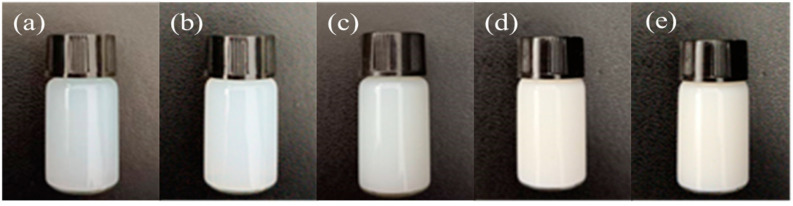
Five types of organic polymer–inorganic nano ZnO core–shell sizing agents after centrifugation at 3000 r/min for 30 min and storage at room temperature for 10 months: (**a**) S1, (**b**) S2, (**c**) S3, (**d**) S4, (**e**) S5.

Storage stability is one of the most important properties of sizing agents. The macroscopic stability of a sizing agent is generally observed using room temperature storage and centrifugation tests. A small amount of the sizing agent was centrifuged at 3000 rpm for 30 min and then stored at room temperature for 10 months. The emulsion state is shown in Figure 4; no sedimentation was observed in the sizing agent emulsions of S1 to S5. These results indicate that the sizing agents with an organic crosslinked polymer core and an inorganic nano ZnO shell structure exhibit good storage stability.

### 3.3. Thermal Properties of Organic Polymer–Inorganic Nano ZnO Core–Shell Sizing Agents

**Figure 5:**
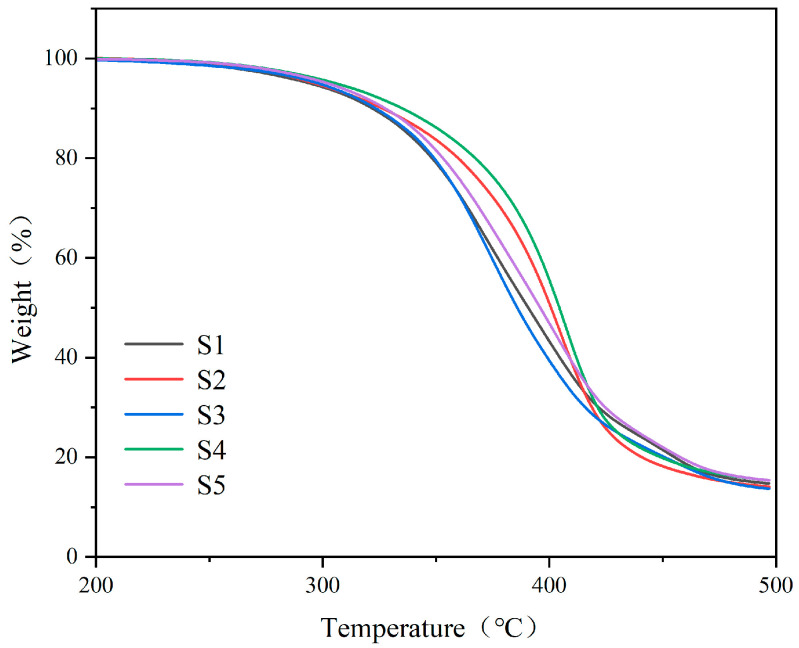
TGA analyses of the sizing agents S1 to S5.

The thermogravimetric analysis (TGA) curves for the organic polymer–inorganic nano ZnO core–shell sizing agents (S1 to S5) are shown in Figure 5. The decomposition temperatures corresponding to 5% and 30% weight loss (T5 and T30) and the thermal stability indices are listed in Table 2 [29]. It can be clearly seen that with an increase in sizing agent MMA content, T5, T30, and the thermal stability indices slightly increase, which is ascribed to the better thermal stability of MMA compared to the amine-modified epoxy resin within the emulsion particles. The TGA results show that all sizing agents exhibit good thermal stability, meeting the processing temperature requirements for CF/EP composites [30].

### 3.4. Surface Morphology and Roughness

**Figure 6:**
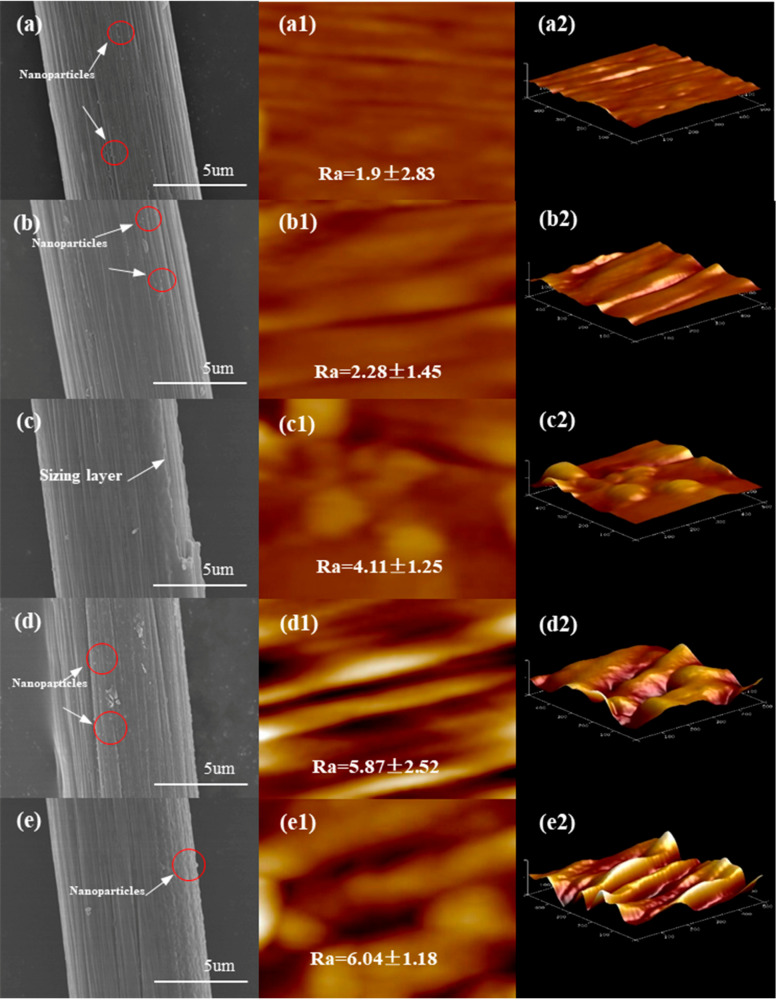
SEM and AFM surface morphology of sized CFs (S1 to S5): surface morphology by SEM (**a**–**e**); 2D height images by AFM and roughness (**a1**–**e1**); 3D height images by AFM (**a2**–**e2**).

The surface morphology of the five sizing agents S1 to S5 applied to the CFs was observed by scanning electron microscopy (SEM). As shown in Figure 6a–e, the grooves on the surface of the carbon fibers are effectively filled after sizing, and the organic polymer–inorganic nano ZnO core–shell particles of the sizing agents increased the surface roughness. The roughness of the fiber surface is denoted by Ra, where a larger Ra value indicates greater surface roughness. The Ra values for the CFs in Figure 6(a1–e1) are 1.9, 2.28, 4.11, 5.87, and 6.04, respectively. The largest Ra value for CF4 in Figure 6(d1) suggests it has the highest surface roughness. The organic polymer–inorganic nano-ZnO core–shell sizing agent maximizes the surface roughness of the sized CF at 50% MMA content, and it then decreases with further increasing MMA. These results are consistent with the effect of the MMA content on the surface energy of the sized CF.

### 3.5. Contact Angle and Surface Energy of the Sized CF

**Figure 7:**
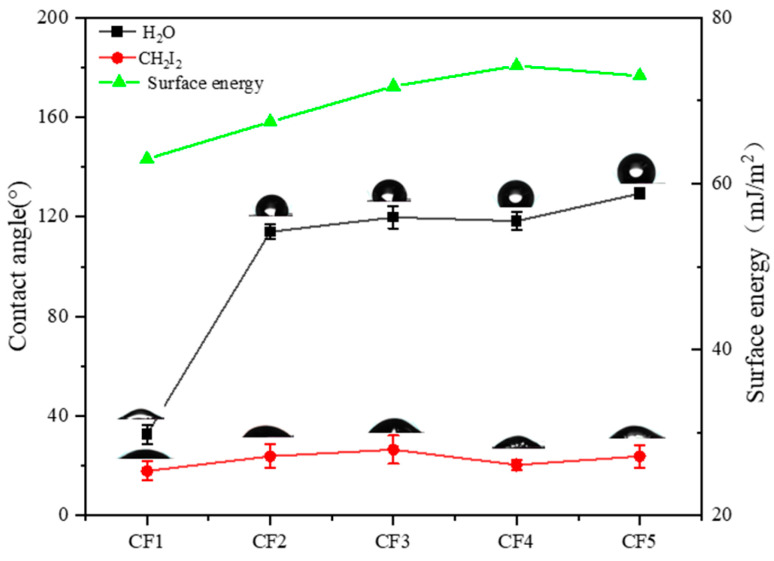
Dynamic contact angles and surface energies of sized CF1 to CF5.

The surface energy of CFs directly determines the mechanical properties of composites. Figure 7 displays the dynamic contact angles of the sized CFs with CH_2_I_2_ and H_2_O. Based on the Owens–Wendt–Rabel–Kaelble (OWRK) and Young’s equations, the surface energy can be derived from the dynamic contact angles with CH_2_I_2_ and water; the testing results for the five types of sizing agents are: 62.79, 67.46, 71.73, 74.21, and 67.97 mJ/m^2^. The CFs sized with S4 display the highest surface energy of 74.21 Jm/m^2^. The particle size of the organic polymer–inorganic ZnO core–shell sizing agent and the surface roughness of the sized CFs increases with MMA, as shown in Figure 3 and Figure 6, so that the surface energy of the sized CFs increases with MMA. These results are consistent with the wetting thermodynamics, in which a higher surface roughness can improve the wettability of hydrophobic solid surfaces via capillary action [31,32], creating the most favorable wetting conditions with dichloromethane. However, the surface energy of CF5 decreased with further increasing MMA; the possible reason is that the particle size of the organic polymer–inorganic ZnO core–shell sizing agent is too big, resulting in poor film-forming properties, and thereby a weaker wetting of CH_2_I_2_ and finally a decreasing surface energy in the sized CFs.

### 3.6. Mechanical Properties of the Nanoparticles in the Organic Polymer–Inorganic Nano ZnO Core–Shell Sizing Agents

**Figure 8:**
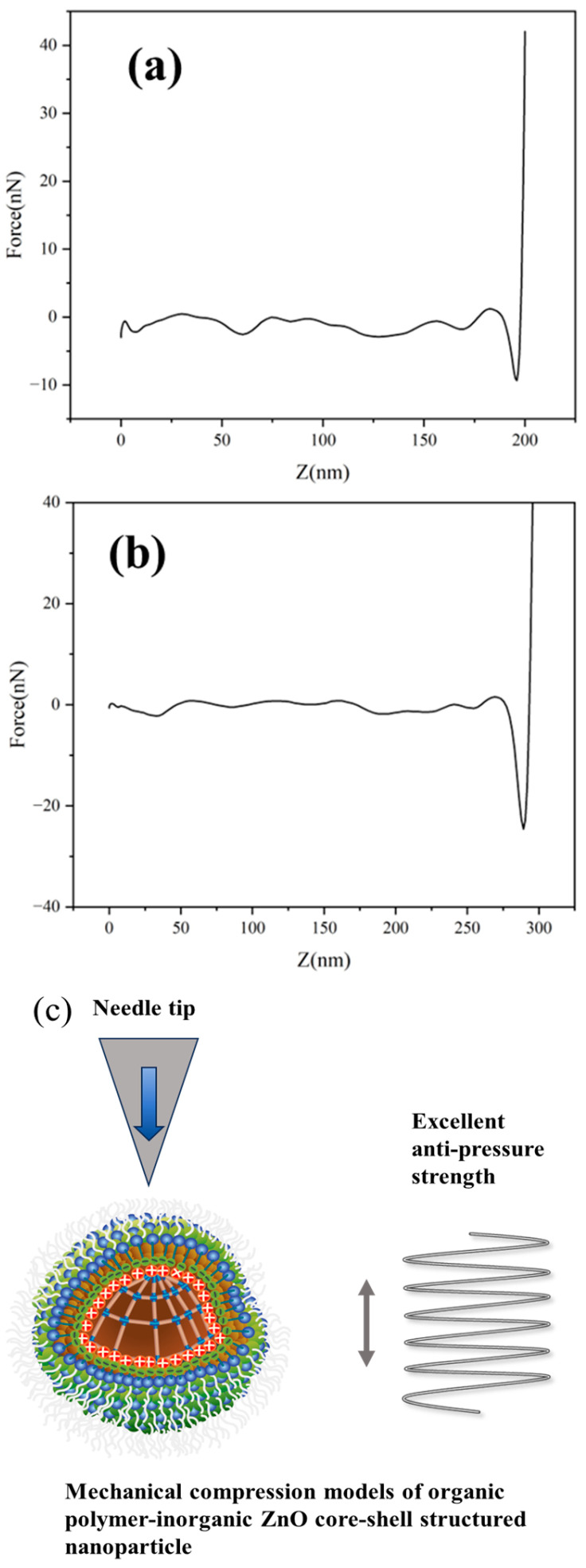
The mechanically continuous anti-pressure performance of the representative sizing agents S0 (**a**), S4 (**b**); Model of a single-particle nanoparticle of a sizing agent under vertical pressure (**c**).

To probe the possible effects of the mechanical anti-pressure properties of the nanoparticles on the stress transfer between the sized CFs and the epoxy (EP) interface, and thereby the CF/EP composite properties, we investigated the mechanical anti-pressure properties of the nanoparticles in the organic polymer–inorganic nano ZnO core–shell sizing agents using AFM Peak Force toolkit measurements (Figure 8) [33,34]. The results showed that the peak force of the nano ZnO particle-modified sizing agent (S0) with a core of non-crosslinked amino modified epoxy resin containing allyl groups was 42 nN. In contrast, the nanoparticle structure of organic polymer–inorganic nano ZnO core–shell sizing agent (S4) exhibited better rigidity with a peak force of 127 nN, due to crosslinking polymerization between the amine-modified epoxy resin containing allyl groups and the MMA inside the core.

Additionally, we also investigated the influence of the MMA content on the crosslinking networks inside the organic polymer core in turn on organic polymer–inorganic nano ZnO core–shell sizing agents to optimize their mechanical performance (Table 3). The amine-modified epoxy resins containing allyl groups are prone to co-polymerization with MMA due to the steric hindrance effect, thereby increasing the crosslinking networks inside the core. However, when MMA increases further, MMA tends to generate a linear polymer which lowers the crosslinking density of the organic polymer inside the core, thereby the anti-pressure performance of the organic polymer–inorganic nano ZnO core–shell sizing agents decreases. In conclusion, we optimized the MMA content to be 50% of the amine-modified epoxy resins containing allyl groups; the sizing agent S4 has the maximum mechanical anti-pressure property to enhance the stress transfer between the CF and the epoxy resin matrix, thus increasing the mechanical properties of the sized CF/EP composites.

### 3.7. Mechanical Properties of CF/ER Composites

**Figure 9:**
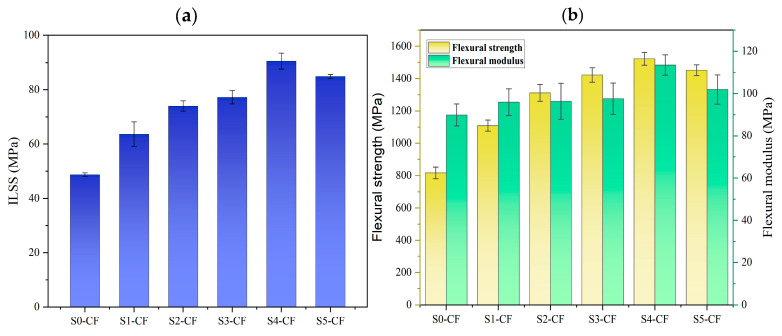
Mechanical properties of the sized CF/ER composites with sizing agents S0 to S5, (**a**) ILSS, (**b**) flexural strength and flexural modulus.

ILSS is an important parameter to evaluate the interfacial adhesion of CF/EP composites [35]. The results shown in Figure 9a indicate that the enhancement of ILSS by the sizing agents prepared in this study did not follow a monotonic trend. S0 represented the sizing agent which was not treated inside the organic polymer core with MMA and served as a control example; the ILSS of the S0 sized CF/EP composite had the lowest value of 48.72 MPa. When the sizing agents were applied on CF from S1 to S5, the ILSS of the sized CF/EP composites significantly increases, and the ILSS of the S4 sized CF/EP material reaches its maximum value, 90.57 MPa. Then, the ILSS of the S5 sized CF/EP composite slightly decreases from the peak value to 84.94 MPa.

The flexural strength and flexural modulus of the sized CF/EP composites from sizing agent S0 to S5 have the same tendency as with their ILSS. The maximum value of both flexural strength and the flexural modulus of the sized CF/EP was achieved with S4, which was improved by 86% and 86.43%, respectively, compared with S0 (Figure 9b). The results indicate that sizing agents S0 to S5 have a significant impact on the mechanical properties of CF/EP composites, while the mechanical anti-pressure properties of the nanoparticles in the sizing agent itself are influenced by the crosslinking density of the organic polymer by the MMA content inside the core. The impact tendency of the mechanical properties of CF/EP composites by sizing agents S0 to S5 coincides with the mechanical anti-pressure properties of the nanoparticles in the sizing agent itself; the nanoparticle in sizing agent S4 has the maximum mechanical anti-pressure properties, and the sized S4 CF/EP composite achieves the maximum mechanical properties.

### 3.8. Fractured Morphology of CF/ER Composites

**Figure 10:**
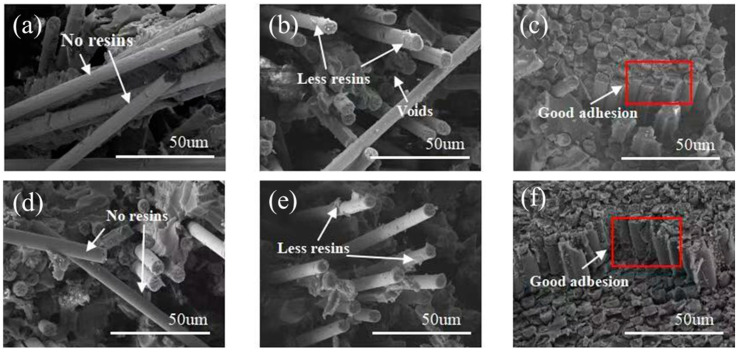
SEM images of the fracture surfaces in the CF/EP composites: (**a**,**d**) CF0/EP; (**b**,**e**) CF1/EP; (**c**,**f**) CF4/EP.

Figure 10 presents the SEM images of the fractured surfaces of the CF0/EP, CF1/EP, and CF4/EP composites. A large number of CFs were pulled out of the resin matrix and left numerous voids when the CF0/EP composite was fractured, with almost no residual resin on the bare CF surface (Figure 10a,d), confirming that the adhesion between CF0 and the EP interface was low [36], and that the mechanical properties of the CF0/ER composite is weak. The fracture morphology of the CF1/EP composite indicates that there are substantial gaps between the sizing layer and the CF surface and only a small portion of the CFs are coated with EP (Figure 10b,e). This suggests that the amine-modified epoxy resins containing allyl groups form a crosslinking polymerization with MMA inside the core that can increase the mechanical anti-pressure properties of the nanoparticles in the organic polymer–inorganic nano ZnO core–shell sizing agent S1 and provide a relatively effective stress transfer at the CF1/EP interface to enhance the mechanical properties of CF/EP composites through a nanoparticle interlocking mechanism on the rough surface [37].

By contrast, the fracture morphology of the CF4/EP composite reveals almost no gaps between the sizing layer and the CF surface. The CFs were tightly arranged by the EP matrix with a flat fracture surface (Figure 10c,f), indicating the outstanding adhesion between the CF surface and the EP matrix, which facilitates force transfer from the EP matrix to the CFs [38]. This is consistent with the tested results of the CF4/EP composite’s mechanical properties, further indicating that the nanoparticles in sizing agent S4 have the maximum mechanical anti-pressure properties, and thereby the most effective stress transfer at the CF1/EP interface and the maximum mechanical properties of the CF/EP composites.

## 4. Conclusions

In this work, organic polymer–inorganic nano ZnO core–shell sizing agents were prepared in situ, and the effect of the crosslinking density of the organic polymer core on the mechanical anti-pressure properties of the nanoparticles was studied; a higher mechanical anti-pressure favors an effective stress transfer at the CF/EP interface for maximum mechanical properties of the composites. The testing results of DLS, TGA, SEM, TEM, AFM, and contact angle measurement have demonstrated that the sizing agents exhibited good particle size uniformity and thermal stability, significantly improving the surface roughness and surface energy of the sized CF and the interfacial wettability of the epoxy resin toward the sized CF. The mechanical properties of the CF/EP composites including ILSS, flexural strength, and flexural modulus were also studied, and the results indicate that the sizing agent S4 achieved optimal performance when the MMA content was 50% of the amine-modified epoxy resin containing allyl groups. The CF/EP composite modified with the sizing agent S4 reached a maximum ILSS of 90.57 MPa, and its flexural strength and the flexural modulus improved by 86% and 86.43%, respectively, compared to the CF/EP composite modified with the sizing agent S0. In the future, the integration of the organic polymer core with inorganic nano ZnO shell through chemical bonding, as well as the surface reaction of sizing agents on CF, will be investigated, and the modified sizing agents will further influence the mechanical properties of the composites.

## Figures and Tables

**Table 1:** The quantity of reactants for the six types of sizing agent emulsion.

Sizing Agents	Quantities of Reactants (g)		
	AEPA	ZAA	MMA
S0	50.21	6.33	0
S1	50.33	6.34	9.79
S2	50.19	6.32	13.02
S3	50.14	6.32	15.60
S4	50.25	6.33	19.55
S5	50.29	6.34	23.48

**Table 2:** Characteristic thermal data for the sizing agents S1 to S5.

Sizing Agents	Characteristic Thermal Data (°C)		
	T_5_	T_30_	T_Heat resistance index_
S1	294	364	165 ± 1.57
S2	297	379	170 ± 1.86
S3	298	363	165 ± 1.49
S4	306	385	173 ± 1.55
S5	302	369	168 ± 1.62

**Table 3:** Elastic modulus for six types of sizing agent emulsion.

Sizing Agent	DTM Modulus (Gpa)
S0	1.04 ± 0.16
S1	4.84 ± 0.22
S2	5.31 ± 0.26
S3	5.54 ± 0.12
S4	6.17 ± 0.15
S5	5.78 ± 0.18

## Data Availability

The original contributions presented in this study are included in the article/Supplementary Materials. Further inquiries can be directed to the corresponding author.

## Supplementary Materials

The following supporting information can be downloaded at: https://www.mdpi.com/article/10.3390/polym17060773/s1, Figure S1: ILSS and flexural strength tests specimen dimensions and instrument: (a) the ILSS test specimen dimensions; (a1) the ILSS test instrument; (b) the flexural strength test specimen dimensions; (b1) the flexural strength test instrument, Figure S2: The particle size and its PDI of S1 to S5, Figure S3: The SEM and TEM images of S1, S2, S3, S5. Figure S4: The SEM and AFM images of CF0.
