# Supplementary material for: In Situ Synthesis of Organic Polymer–Inorganic Nano ZnO Core–Shell Structured Sizing Agents and Their Effect on Carbon Fiber Interfaces and Composite Properties
Source: Polymers (Basel). 2025 Mar 14;17(6):773. doi: 10.3390/polym17060773 (PMC11944867; doi:10.3390/polym17060773)
Supplement: Supplementary file 1 [file polymers-17-00773-s001.zip › polymers-3481139-supplementary.pdf]

Supplementary Information

In-situ synthesis of organic polymer-inorganic nano ZnO core-shell structured sizing agents and their effect on carbon fiber interfaces and composite properties

Wen Liu [1], Mudasir Ahmad [2], Pengfei Song [1], Qianli Fang [1], Qingchao Li [1], Guoqing Huang [1,*] and Chuncai Yang [1,3,*]

1 Institute of Catalysis for Energy and Environment, College of Chemistry and Chemical Engineering, Shenyang Normal University, Shenyang 110136, China; 18241394119@163.com (W.L.); 18698785870@163.com (P.S.); 17863521569@163.com (Q.F.); 15945315056@163.com (Q.L.);
2 School of Chemistry and Chemical Engineering, Northwestern Polytechnical University, Xian 710072, China; mirmudasirv@nwpu.edu.cn
3 Jilin Qianren Innovative Materials Co., Ltd., Jilin 132101, China
* Correspondence: hgq3917@163.com (G.H.); ccy_0823@163.com (C.Y.)

1.Mechanical testing of CF/EP composites

1.1 Interlaminar shear strength （ILSS）

The interlaminar shear strength of CF/EP composites was tested in the force range of 0~10000 N using a Instron 34TM-30 universal tensile tester, according to ISO14130:1997(E). The loading rate was set to 1 mm/min. The ILSS test was performed using the short beam method Fig.S1a-a1 shown. The CF/EP composite specimens were placed on the apparatus, and the averaged values of 5 times were measured. ILSS was calculated as below.The formula is as equation .

$$ILSS = \frac{3P}{4bh}$$

ILSS(MPa) is interlaminar shear strength of CF/EP composites, P is the maximum load on the specimen before fracture (N), and b(mm) and h(mm) are the width and thickness of the specimen, respectively.

1.2 Flexural strength

Academic Editor: Gregorio Cadenas-Pliego

Received: 31 January 2025
Revised: 12 March 2025
Accepted: 13 March 2025
Published: date

Citation: Liu, W.; Ahmad, M.; Song, P.; Fang, Q.; Li, Q.; Huang, G.; Yang, C. **In** Situ Synthesis **of** Organic Polymer–Inorganic Nano **ZnO** Core–Shell Structured Sizing Agents **and** Their Effect **on** Carbon Fiber Interfaces **and** Composite Properties. *Polymers* **2025**, *17*, x. https://doi.org/10.3390/xxxxx

Copyright: © 2025 by the authors. Submitted for possible open access publication under the terms and conditions of the Creative Commons Attribution (CC BY) license (https://creativecommons.org/licenses/by/4.0/).

The bending strength of CF/EP composites was measured in the force range of 0~10000 N as shown in Fig S1b-b1 using Instron 34TM-30 universal tensile tester according to ISO 14125:1998(E) year. The bending strength was calculated using the following equation.

$$\sigma_f = \frac{3FL}{2bh^2}$$

Where σf is the Flexural strength (MPa), F is the maximum load on the specimen before fracture (N), b(mm) and h(mm) are the width and thickness of the specimen, respectively. L is the spacing between the two supports.

1.3 Flexural modulus

The flexural modulus of CF/ER composites was measured in the force range of 0~10000 N using Instron 34TM-30 universal tensile tester in accordance with ISO 14125:1998(E) year. The bending modulus (Ef) was calculated using the following equation.

$$E_f = \frac{L^3}{4bh^3}\left(\frac{\Delta F}{\Delta S}\right)$$

Where Ef is the flexural modulus (MPa), ΔF is the amount of load increase with the initial linear portion of the load-displacement curve (N), ΔS is the increment of the displacement corresponding to ΔF, L is the spacing between two supports, and b, h(mm) and Fmax are the width, thickness and maximum load of the test specimen, respectively.

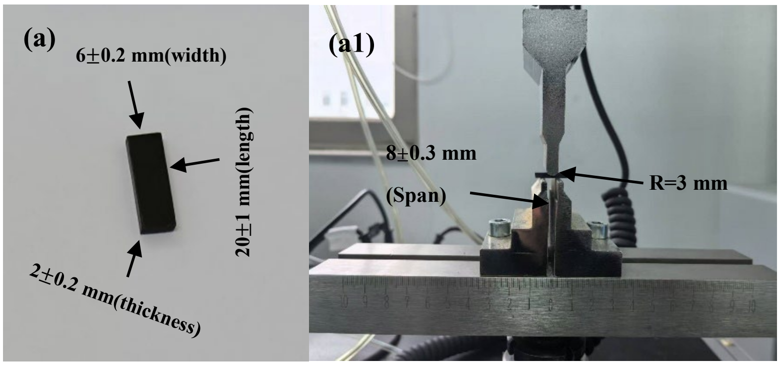

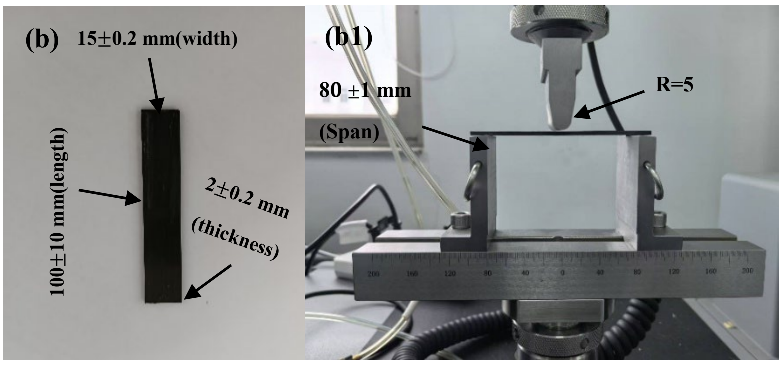

Figure S1. ILSS and flexural strength tests specimen dimensions and instrument: (a) the ILSS test specimen dimensions; (a1) the ILSS test instrument; (b) the flexural strength test specimen dimensions; (b1) the flexural strength test instrument

1.4 The Owens-Wend-Rabel-Kaelble (OWRK) and Young' equation

The Young's equation is the theoretical basis for all surface free energies, and the Young's equation is as follows:

$$\gamma_{sl} = \gamma_s + \gamma_l - 2\phi(\gamma_s\gamma_l)^{0.5}$$

γ_s is the surface free energy of a solid, γ_l is the surface free energy of a liquid, γ_{sl} is the surface free energy between solids and liquid.

The formula for the OWRK equation is:

$$\sqrt{\gamma_{sv}^d\gamma_{lv}^d} - 2\sqrt{\gamma_{sv}^P\gamma_{lv}^P} = 0.5\gamma_{lv}(1 + cos\,\theta_Y)$$

Due to the presence of two unknowns in the equation, γ_{sv}^d and γ_{sv}^P, two liquids with known dispersion and polarity components are required. One type of liquid requires the selection of a liquid primarily composed of polar components, while the other type. The liquid selection is mainly for the dispersion part of the liquid. This is because of mutual interaction. It occurs between similar components. If we only have the dispersion part. Possible polarity interactions will be ignored. H_2O and CH_2I_2 are the most common choices.

1.5 The histogram of DLS measurements

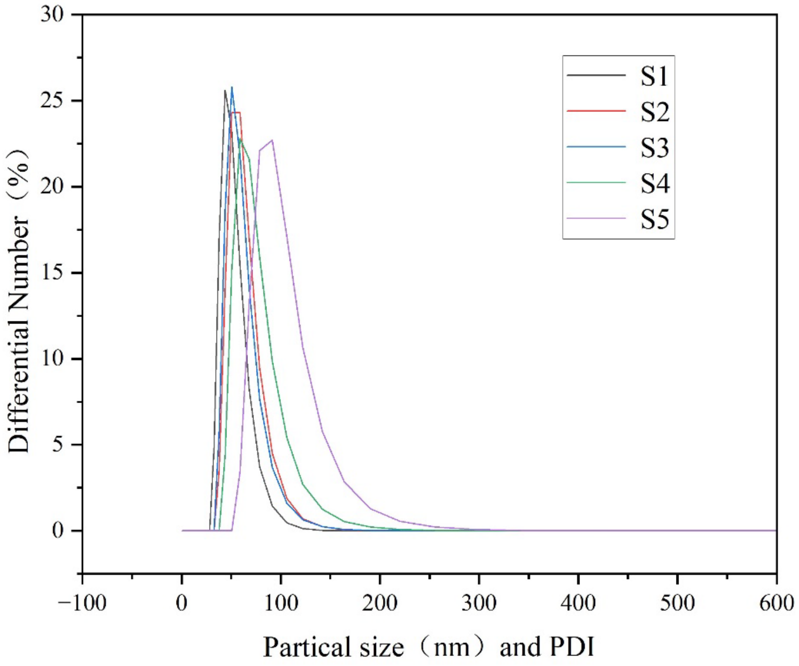

Figure S2. The particle size and its PDI of S1 to S5.

From the Figure S2, it can be seen that all five types sizing agents are nanoscale sizing agents.

1.6 SEM and TEM images of S1, S2, S3, S5

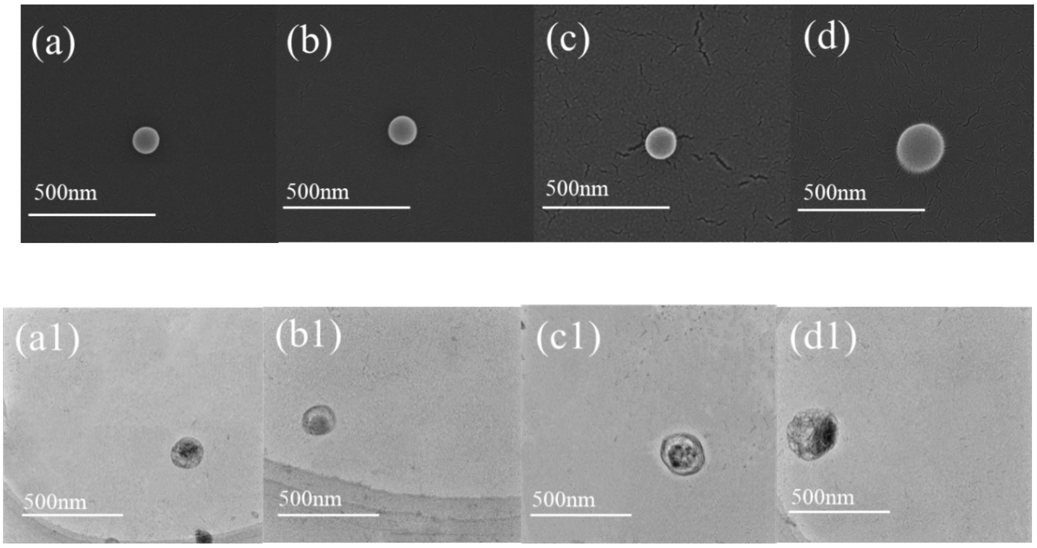

Figure S3. The SEM and TEM images of S1, S2, S3, S5.

All sizing agents observed from Figure 3 have successfully synthesized core-shell structures and meet the particle size requirements in the particle size test results.

1.7 SEM and AFM images of CF0

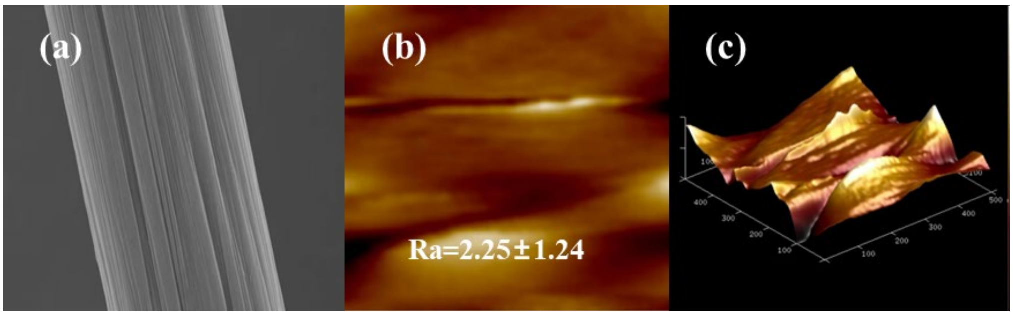

Figure S4. The SEM and AFM images of CF0.

As shown in Figure S4, the SEM(a) and AFM2D(b), 3D(c) images of carbon fiber after sized with modified sizing agent are presented. From (a), it can be seen that after desizing treatment, there is basically no residue of slurry on the surface of carbon fiber, and there are obvious grooves and grooves on the surface. Therefore, its roughness reaches 2.25nm. After sizing, the surface defects of carbon fiber are effectively filled.
